# Microglia Mediated Neuroinflammation: Focus on PI3K Modulation

**DOI:** 10.3390/biom10010137

**Published:** 2020-01-14

**Authors:** Antonia Cianciulli, Chiara Porro, Rosa Calvello, Teresa Trotta, Dario Domenico Lofrumento, Maria Antonietta Panaro

**Affiliations:** 1 Department of Biosciences, Biotechnologies and Biopharmaceutics, University of Bari, 70125 Bari, Italy; antonia.cianciulli@uniba.it (A.C.); rosa.calvello@uniba.it (R.C.); 2 Department of Clinical and Experimental Medicine, University of Foggia, 71100 Foggia, Italy; chiara.porro@unifg.it (C.P.); teresa.trotta@unifg.it (T.T.); 3 Department of Biological and Environmental Sciences and Technologies, Section of Human Anatomy, University of Salento, 73100 Lecce, Italy; dario.lofrumento@unisalento.it

**Keywords:** neurodegeneration, inflammation, PI3K, microglia, signaling pathway

## Abstract

Immune activation in the central nervous system involves mostly microglia in response to pathogen invasion or tissue damage, which react, promoting a self-limiting inflammatory response aimed to restore homeostasis. However, prolonged, uncontrolled inflammation may result in the production by microglia of neurotoxic factors that lead to the amplification of the disease state and tissue damage. In particular, specific inducers of inflammation associated with neurodegenerative diseases activate inflammatory processes that result in the production of a number of mediators and cytokines that enhance neurodegenerative processes. Phosphoinositide 3-kinases (PI3Ks) constitute a family of enzymes regulating a wide range of activity, including signal transduction. Recent studies have focused attention on the intracellular role of PI3K and its contribution to neurodegenerative processes. This review illustrates and discusses recent findings about the role of this signaling pathway in the modulation of microglia neuroinflammatory responses linked to neurodegeneration. Finally, we discuss the modulation of PI3K as a potential therapeutic approach helpful for developing innovative therapeutic strategies in neurodegenerative diseases.

## 1. Introduction

Neurodegeneration, a slow and progressive destruction of neuronal cells, represents the pathological condition common to several brain disorders, including Alzheimer’s disease (AD), Parkinson’s disease (PD), multiple sclerosis (MS), Huntington’s disease (HD), and amyotrophic lateral sclerosis (ALS) [1,2].

Among the many cellular and molecular processes responsible for neurodegenerative events, such as oxidative responses, accumulation of protein aggregates, altered mitochondrial function, and triggering of apoptosis [2], neuroinflammation is correlated with the onset and progression of a number of neurodegenerative diseases of both an acute and chronic nature [3,4,5]. In general terms, neuroinflammation is a defense mechanism aimed to protect the central nervous system (CNS) in response to a variety of insults, including infection, traumatic injury, toxic metabolites, or autoimmune events [6]. Although it is believed that an acute neuroinflammatory response is generally gainful to the CNS, minimizing further injury and contributing to the tissue homeostasis, chronic neuroinflammation in the CNS can give rise to severe damage to the neuronal compartment, interfering with its homeostatic integrity, ruffling the balance between reparative responses and pro-inflammatory events [7].

Multiple studies suggest the involvement of microglia as a critical executor of the inflammation mediated neurodegeneration. Microglia, the resident immune cells of the CNS, play a defense role in the CNS against potentially dangerous agents, although recently, an emerging role has been described concerning their involvement in the prompting of pathological processes. Active microglia have beneficial functions, promoting pathogen clearance and tissue regeneration, contributing to neuronal survival through the release of beneficial trophic factors [8]. Interestingly, microglia are multifaceted cells since they may change their phenotype and function during neurodegenerative disorders, such as PD, AD, MS, ALS, stroke, and traumatic brain injury (TBI), eliciting pro-inflammatory responses [8].

If left unchecked, during brain damage or infection, activated microglia prompt and sustain an inflammatory phenotype, releasing a variety of neurotoxic molecules, ultimately leading to neuronal demise [9]. Although recent lines of evidence attributed to microglia a common disease associated signature [10,11,12], the mechanisms regulating the microglial phenotype in neurodegenerative processes have not yet been clarified.

Phosphoinositide 3-kinases (PI3Ks) regulate several key mechanisms in the inflammatory response to external insults [13]. PI3Ks are enzymes able to transduce signals deriving from growth factors, cytokines, hormones, as well as lipopolysaccharide (LPS) into intracellular messages, thus generating phospholipids. These, in turn, lead to the serine/threonine kinase Akt and other downstream effector pathways. Eight PI3Ks are described in mammalian cells and classified into three families [14], differing in their regulation and preferred lipid substrate. The PI3K lipid kinases are involved in many cellular functions, including signal transduction, as well as intracellular vesicular traffic, then dysregulation of PI3K pathways is involved in a number of pathological conditions such as neurological diseases. Experimental evidence indicated that inhibition of the PI3K/Akt pathway in the LPS activated microglia leads to a reduced level of proinflammatory factors [15]. Conversely, another study reported that PI3K/Akt activation contributes to the attenuation of brain damage, being able to upregulate anti-oxidative response with the inhibition of inflammation and apoptosis [16]. Therefore, these results point out that there are diversified effects mediated by this crucial signal molecule in orchestrating both protective and possibly harmful responses.

Considering the important role of neuroinflammatory processes in the outbreak of neurodegenerative pathologies, this review highlights PI3K as a key actor for signaling pathways in the inflammatory responses involved in neurodegeneration. The search for potential PI3K modulators provides possible targets for anti-inflammatory therapies able to slow down or counteract the progress of neuroinflammation.

## 2. PI3K Signaling Pathway

PI3Ks constitute a conserved family of kinases able to phosphorylate one or more inositol phospholipids in the 3-position present on the inositol ring, generating other molecules such as phosphatidylinositol-3,4,5-trisphosphate (PtdIns(3,4,5)P3,phosphatidylinositol-3,4-bisphosphate (PtdIns(3,4)P2), and phosphatidylinositol-3-phosphate (PtdIns(3)P) [14,17]. These PI3K enzymes are located on the inner side of the plasma membrane where they propagate intracellular signaling cascades regulating a wide range of actions that comprise signal transduction, vesicular traffic, and cytoskeletal reorganization [14,18].

### 2.1. Classes of PI3K Enzymes

In mammalian cells, the PI3Ks can be divided into three classes (I, II, and III) according to substrate affinity, functional homologies and their structural characteristics. Class I PI3Ks are composed of two subtypes, classes IA and IB, based on different associated adaptors [19].

The heterodimers of class IA consist of a catalytic p110 and a p85 regulatory subunit. The p110 isoforms include three different types (110α, p110β, and p110δ), whereas there are five regulatory p85 polypeptides (p85α, p85β, p55α, p50α, and p55γ) [20,21]. The p110δ isoform is mostly confined to expression in immune cells, whereas the p110α and β isoforms are expressed in all cell types [22]. The p85α and β regulatory subunits are expressed in many cells, whereas the other polypeptides are distributed in a few tissues (see Table 1).

The p110 subunits contain five regions: a C-2 region for membrane anchoring, a Ras binding region (RBD), an N-terminal region called the adaptor binding region (ABD) that interacts with the regulatory p85 family members, a helical region, and a kinase catalytic region. The regulatory subunits share three core regions, including a p110 binding region called the inter-Src-homology-2 (iSH2) region, flanked by two SH2 regions that bind constitutively to the p110 ABD. In addition, p85α and p85β have a Rho GTPase activating protein (GAP) region and N-terminal portions containing an SH3 region. The SH2 regions in p85 recognize the same phosphotyrosine motif and allow the enzyme to associate with activated tyrosine kinase receptors (RTKs) and other intracellular phosphotyrosine containing proteins [13,20].

Class IA enzymes are stimulated by cytokines, receptor tyrosine kinases (RTKs), G protein coupled receptors (GPCRs), and the small G protein Ras [33]. These receptors recognize interleukin-6 (IL-6), hormones, epidermal growth factors (EGF), insulin-like growth factors (IGF), and inflammatory stimuli such as LPS, and the cellular signals known to be modulated comprise growth, survival, cell proliferation, and inflammatory processes [17,34].

Class IB enzymes are dimers of the p110γ polypeptide complexed with an adaptor subunit, p101 or p84 and p87 [28,35]. Subunit p101 or p84 bind to a single p110γ polypeptide to obtain two heterodimers, p101/p110γ or p84/p110γ, both known as “PI3Kγ” [14,29,36]. PI3Kγ is expressed in both tissues and cells, with the highest levels of distribution in myeloid cells [37,38]. Differently from class IA catalytic subunits, p110γ contains a Gβγ binding domain in the catalytic region at the C-terminal, and it is mainly stimulated by GPCRs, thanks to the interaction of its the regulatory subunit with the subunit of trimeric G protein and therefore distinct from class IA PI3Ks, which instead are activated downstream of RTKs [39].

Each of the Class I PI3K isoforms catalyzes the phosphorylation of PtdIns (4,5)P2 to generate PtdIns (3,4,5)P3, known as “PIP3”, and the substantial difference between them appears to be in their adaptation to upstream regulation by receptor transduction pathways [14,29,36].

Class II PI3Ks have only one catalytic protein present in three different isoforms (PI3K-C2α, PI3K-C2β, and PI3K-C2γ), which preferentially uses PtdIns or PtdIns4P as substrates at the plasma membrane or endosomes, therefore forming both PtdIns3P and PtdIns (3,4)P2 [30,40]. These enzymes lack the regulatory-subunit binding region, but possess a Ras binding region and the PI3K core. Distinctively, they all possess a C-terminus extension consisting of a Phox homology (PX) region, a second C2 region, and several additional protein regions at their N-terminal region [30].

Many studies show that several stimuli can activate these isoforms, including hormones [30,41], chemokines such as monocyte chemotactic protein 1 (MCP1), cytokines such as tumor necrosis factor α (TNFα) [42], growth factors such as EGF and stem cell factor (SCF) [43], integrins [44], and phospholipids such as lysophosphatidic acid (LPA) [45].

These studies show that only some class II PI3K isoforms can be triggered downstream of RTKs [30,43] and GPCRs [45]. The cellular activity of Class II PI3K remains poorly characterized with respect to class I kinases. Furthermore, the substrate used by these enzymes is PtdIns4P [30].

Lastly, class III PI3K consists of a single catalytic subunit termed Vps34 (vacuolar protein sorting 34), and its activity is modulated by the Vps15 kinase (known as p150 in mammalian cells) with which it binds [46]. Vps34 phosphorylates PtdIns to form PtdIns3P with certain intracellular structures such as endosomes and, through binding to distinct effector regions, modulates the activity and fate of these vesicular structures [47].

Vps34 is a lipid kinase that mediates cell signaling through mammalian target of rapamycin (mTOR), indicating a possible role in modulating cell growth [47].

### 2.2. PI3K Signaling Pathway Activation

The typical activation manner of one or more isoforms of PI3Ks enzymes begins with the binding of a ligand to RTKs or GPCRs through a regulatory subunit, such as p85. In basal conditions, p85 associates with the N-terminal of the catalytic p110 subunit through its iSH2 region, inhibiting its function. Following appropriate cellular stimuli as many cytokines and growth factors, as well as by insulin and LPS [14], the SH2 regions bind to activated receptors or adapter proteins, and this phosphotyrosine binding leads to allosteric activation of the p110 catalytic subunits of PI3K [33,48]. In particular, the activation of class I PI3Ks converts the cellular membrane phospholipid PtdIns (4,5)P2 to PIP3 [14,48], whereas PtdIns (3,4)P2 can originate from PIP3 through the SHIP family of phosphatases (SHIP-1 and SHIP-2) [18,49,50]. In addition, the class II PI3Ks using PI4P as a substrate can also produce PI3,4P2 [17]. Although the activation of specific PI3K isoform in a given cellular process can be different, the ultimate result is the same: repositioning and activity of numerous signaling proteins through binding to conserved regions like the PH (pleckstrin-homology) regions.

Therefore, PIP3 or PI3,4P2 as second messengers function as ligands to call back the PH region containing proteins at the inner side of the plasma membrane. These proteins including Akt (protein serine/threonine kinase) (also termed PKB) and PDK1 (phosphoinositide dependent kinase 1), which share a PH region selective for PIP3 and/or PtdIns 3,4-P2 [29]. Akt consists of three isoforms (Akt1, Akt2, and Akt3), which share a similar architecture: an N-terminus PH region, a central serine/threonine catalytic region, and a small C-terminus regulatory region. The PH region is critical for the PI3K/Akt signaling transduction [51].

**Figure 1:**
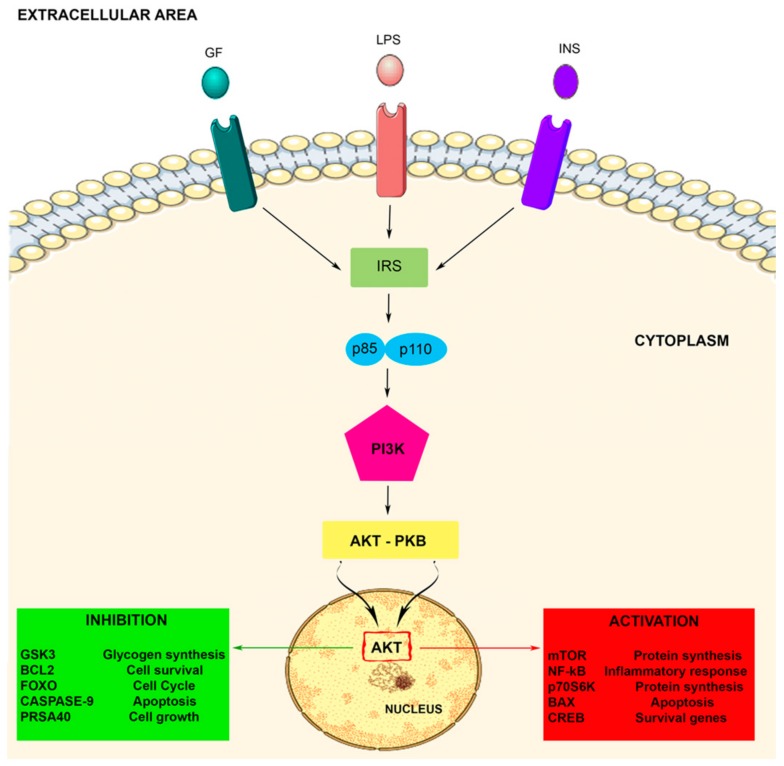
The PI3K-Akt signaling pathway. The PI3K-Akt pathway is involved in some crucial cellular processes including protein synthesis and cell proliferation and survival. The PI3K/Akt pathway is activated by factors that initiate the PI3K signaling pathway via intermediate molecules (IRS), playing an important regulatory role in many cellular survival pathways. The pathway can be activated by a variety of signals, including growth factors (GF), LPS, and insulin (INS), targeting several downstream molecules. This activation is able to modulate cell activities, including cell proliferation, glucose metabolism, cell survival, cell cycle, protein synthesis, and neuronal morphology and plasticity.

Akt activation allows the movement of the protein kinase towards the cytoplasm and nucleus, where it modulates numerous downstream proteins such as the Bcl-2 antagonist of cell death, caspase 9, glycogen synthase kinase-3 beta (GSK3β), p70 S6 kinase (p70S6K), fork head transcription factors (FOXOs), proline rich Akt substrate of 40kDa (PRAS40), nuclear factor-kappa B (NF-kB), and mTOR, either positively or negatively. These target proteins are implicated in several cell biological activities such as protein synthesis, cell proliferation, cell motility, metabolism, survival, apoptosis, cell growth, and neuroinflammatory disorders [33,48,51]. Therefore, Akt represents a major mediator of PI3K signals, and the PI3K/Akt signaling pathway plays important roles in the cellular responses (see Figure 1).

## 3. Role of PI3K in Microglia Activity

It has been well established that neuroinflammation is an intricate inflammatory reaction that may occur within the peripheral nervous system or in the CNS, being generally triggered by tissue injury and infectious agents. It is an extremely dynamic and complex adaptive process, which definitely has the purpose of containing cellular damage, maintaining brain tissue integrity, as well as restoring tissue homeostasis. The production of pro-inflammatory cytokines in glial cells, especially in microglia and astrocytes, represents an intersection point between the immune and nervous systems, assigning to microglia the role of the predominant supplier of inflammatory mediators in the CNS [52].

Microglia, astrocytes, and neurons, as well as all the CNS resident cells actively participate in neuroinflammation: among all, microglia and astrocytes seem to contribute extensively, playing a pivotal role in neuroinflammatory response regulation [53].

Microglia and astrocytes are therefore actually believed to be responsible for the innate immune response in CNS, expressing a defense function against pathogens and damage. In this regard, even if reactive astrocytes seem able to coincide with neuroinflammation, after activation of microglia and neural injury, microglia remain, without any doubt, the most important component of neuroinflammation [54].

In this regard, it is well understood that microglia scan the microenvironment continuously by generating factors that influence astrocytes and neuron that are in close proximity, triggering an inflammatory response that further includes a temporary, self-limiting response through the immune system and initiates tissue repair. During pathological conditions, when normal resolution mechanisms fail, an irregular activation and development of inflammatory factors results in a chronic neuroinflammatory state [55].

It has been shown that microglia can either activate neuroinflammatory pathways that can lead to a progressive neurodegeneration or alternatively can promote neuroprotection by downregulation of inflammation and stimulation of neuronal repair. Therefore, microglia should not be considered as a uniform entity, but rather as a heterogeneous community with sub-populations that can be recognized on the basis of their different abilities to operate and perform various functions [56].

In particular, a microglial polarization process is well recognized, suggesting the existence of two different forms of microglial cell response, one associated with neuroinflammation, while the other associated with neuroprotection. Moreover, it seems that microglial cells polarization process involves the microglial receptors leading to cellular signal transduction through a variety of intracellular pathways, such as MAPKs, STATs, PI3K, and NF-κB. However, to date, knowledge about microglial cell polarization during neuroinflammation is still limited, and it is the result of a restrained number of recent studies concerning stroke, depression, neuroinflammation, or PD [34].

In this context, recent studies have focused substantial attention on the intracellular pathway that involves PI3K and the Akt kinase (PI3K/Akt). In addition, an important role of the PI3K/Akt cascade in the functions of microglial cells was recently reported. The activation of this signaling cascade is initiated by many cytokines and growth factors, as well as LPS, suggesting that the PI3K/Akt signaling pathway achieved via its specific receptor is crucial in LPS induced inflammation, playing a significant role in the microglial activation pathway, as well as in neuroinflammation [15]. As a consequence, regulation of the PI3K/Akt axis is to be considered a possible approach in order to develop new treatments for neuropathological disorders by the inhibition of proinflammatory gene expression.

In line with this is the observation that curcumin seems to be able attenuate the release of the proinflammatory mediators in BV-2 microglia stimulated with LPS. This may be possible thanks to the suppression of the NF-κB pathway, obtained by the PI3K/Akt signaling pathway downregulation, which could cause a restriction of LPS induced inflammatory responses [57].

It seems that in CNS, the PI3K/Akt pathway can represent an important signaling for neuroprotection. Nevertheless, some possible cytodynamics involving PI3K/Akt and its relation to progressive neurodegenerative disease remain an area of intense interest for research on neuroinflammation and related diseases. Moreover, it has become increasingly evident that several active biomolecules seem able to target the impaired PI3K/Akt balance and, for this reason, could significantly contribute to neuroprotection. This is extremely important in terms of longevity and could represent a promising target for therapeutical strategies improvement [58].

## 4. PI3K Role in Neuroinflammation

Following stimulus, microglia and astrocytes show distinct temporal activation patterns: microglia usually are the first cell population that responds to CNS homeostatic alteration; astrocytes are sequentially activated by microglia. Once activated, astrocytes can modulate the recruitment and activation of other immunocompetent cells such as microglia to the injury site in order to resolve the inflammatory process [59].

In neurodegenerative diseases, the innate immune response has a prominent role compared to adaptative immunity. In particular, microglia trigger typical innate immune responses by enhancing the expression of TLRs and the expression of several pro-inflammatory mediators (TNF-α, IL-1β, IL-6). The TLRs activated PI3K/Akt microglial pathway induces an increase in pro-inflammatory factors production. In these conditions, microglia acquire a neurotoxic phenotype, produce reactive oxygen species, nitric oxide, proteases, and proinflammatory cytokines such as IL-6, IL-1β, IL-12, and TNF-α, attracting more glial cells, which accelerate neuronal damage [60]. In physiological conditions, inducible NO synthase (iNOS) is not expressed in the brain, but its expression is induced in microglia and astrocytes by inflammatory mediators such as LPS and cytokines [61]. Following its expression, high levels of NO are continuously produced, leading to neuronal damage by the inhibition of cytochrome oxidase [62].

ROS and reactive nitrogen species interact with specific amino acid residues on tyrosine phosphatases, protein tyrosine kinases, and protein kinase C that can activate kinase cascades, MAPKs, and PI3Ks. Once activated, PI3Ks in turn phosphorylate phosphatidylinositol-4,5-bisphosphate to phosphatidylinositol-3,4,5-trisphosphate, which is a signaling molecule able to recruit phosphoinositide dependent kinase-1 and Akt. Phosphatase and tensin homolog (PTEN) can downregulate the PI3K/Akt signaling pathway; nevertheless, H_2_O_2_ can oxidize and inhibit PTEN, leading to enhanced activation of the PI3K/Akt signaling pathway. Akt plays a key role in many cellular activities, such as the activation of NOX, leading to additional ROS generation [63].

Recently, it was evidenced that the vulnerability of dopaminergic (DA) neurons could be related to metabolic stress, resulting from the alteration of signaling pathways, including PI3K/Akt. In fact, many biomolecules have been discovered that can activate/modulate the PI3K/Akt pathway in order to reduce the degeneration of neurons by the destructive effects of p38 MAPK activation. This process, in which the activated Akt interacts with JNK, FoxO, GSK3β, and other mediators, elicits neuroprotection by preventing ROS production by activated microglia, thus keeping oxidative stress levels under check, leading to the reduction of neuronal apoptosis [64].

In another study, aimed to unravel the role of the PI3K/Akt pathway in microglia, the molecular mechanism was demonstrated underlying the protective role of benfotiamine, a derivative of thiamine monophosphate, in LPS activated BV-2 microglia. This molecule inhibits microglial activation by blocking the PI3K/Akt, ERK1/2, and JNK signaling pathways, leading to the reduction of NO production and of iNOS, Cox-2, and Hsp 70 expression, as well as of TNF-α and IL-6 [65].

In cultured microglia, activation can be induced by inflammatory stimuli, such as LPS [66]. In an inflammatory environment, activated microglia acquire the macrophage-like phenotype, which triggers an inflammatory cascade, leading to chronic inflammation, which is associated with several neurodegenerative diseases. IL-10 modulates the PI3K pathway and inhibits the LPS induced production and release of IL-1β and TNF-α, suggesting that PI3K is involved in the activation of inflammatory responses in microglial cells. Therefore, the PI3K/Akt pathway can be involved in the activation of NF-κB, leading to elevation of pro-inflammatory mediators in BV2 cells [15,67].

The investigation of the possible role of the PI3K/Akt signaling pathway in neuroinflammation has been conducted essentially in the last decade.

In this respect, some authors demonstrated, in mesencephalic neuron-microglia mixed cultures and microglia enriched cultures treated with pioglitazone and/or LPS, that pioglitazone was able to inhibit LPS induced NO production via increasing PI3K/Akt, thus protecting DA neurons against LPS insult. Therefore, from this study it emerged that the PI3K pathway actively participates in the negative regulation of LPS induced NO production, thus suggesting that the PI3K/Akt pathway plays a role in the regulation of the neuroinflammatory process [68].

In primary cortical and mesencephalic neuron-glia cultures, it has been shown that clozapine was able to reduce inflammation related neurodegeneration induced by LPS. Pretreatment of cortical or mesencephalic neuron-glia cultures with clozapine, in fact, was shown to attenuate LPS induced neurotoxicity. This neuroprotective effect via the attenuation of microglia activation was probably due to a reduction of PHOX generated ROS through inhibiting the PI3K signaling pathway [69].

IRF3 is a transcription factor required for the induction of IFN-γ expression following TLR3 or TLR4 activation. It has been shown, in primary human microglial cultures, that adenovirus mediated IRF3 transgene expression changes the microglial phenotype from pro-inflammatory M1 to anti-inflammatory M2 by modulating the PI3K pathway, suggesting that it plays a predominantly anti-inflammatory role in microglial activation. In particular, it has been demonstrated that PI3K induces the expression of anti-inflammatory and immunoregulatory cytokines such as IL-10, IL-1ra and IFN-γ, suggesting that activation of the PI3K/Akt pathway in microglia can lead to the resolution of neuroinflammation and promotion of tissue repair [70].

A further study proposing a protective role by the PI3K/Akt pathway was carried out in primary microglial cells pretreated with lithium and stimulated with LPS. In this study, Dong and colleagues [71] demonstrated that lithium significantly inhibited LPS induced microglial activation and pro-inflammatory cytokine production through the activation of PI3K/Akt signaling. In particular, the lithium pretreatment led to the suppression of LPS induced TLR4 expression in activated microglia by triggering the PI3K/Akt/FoxO1 signaling pathway. These results confirmed that the PI3K/Akt pathway could have a protective role in LPS induced microglial activation [71].

In addition, it was demonstrated that well established immunosuppressive drug 6 mercaptopurine (6-MP) was able to downregulate microglial inflammatory responses by modulating microglial TNF-α production, suggesting the possible mechanism of this modulation [72]. In this study, in which once again LPS was used to induce inflammatory responses in cultured primary microglia or murine BV-2 microglial cells, it was reported that 6-MP was able to adverse TNF-α mRNA translation through prevention of LPS activated PI3K/Akt/mTOR signaling cascades. These results corroborated the idea that the modulation of PI3K/Akt/mTOR signaling might contribute to the downregulation of microglia mediated inflammation, suggesting its possible therapeutic application in neuroinflammation related neurodegenerative disorders [72].

Therefore, as illustrated by the most recent literature, in microglia, the PI3K/Akt dependent signaling pathway is involved in the expression and production of pro-inflammatory mediators [71], thus gaining a role of primary importance in the modulation of neuroinflammation.

In this regard, many authors in the last few years have reported that there are several substances of natural origin, as well as of a synthetic nature with the ability of inhibiting, both in vitro and in vivo, the PI3K/Akt inflammatory signaling pathway and, consequently, reducing the microglia activation and the inflammatory responses associated with this pathway. A number of in vitro models, including microglia cell lines, stem cell derived microglia cultures, and primary dissociated cell cultures, were performed in order to study the role of microglia in neurodegenerative disorders and, in particular, relevant to PI3K signaling (for more detail, see [73]). This anti-inflammatory beneficial effect could be linked to the ability of inhibiting PI3K/Akt signaling pathways [74,75].

## 5. Therapeutic Strategies on the Modulation of PI3K Signaling

The PI3K pathway is involved in many common diseases, such as cancer, diabetes, cardiovascular disease, and neurological diseases [48]. In particular, its dysregulation has a role in brain pathologies such as developmentally associated brain malformations [76,77], epilepsy [78], aging associated neurodegeneration [79] and brain cancer [80].

In neurodegenerative diseases, dysregulation of PI3K/Akt signaling leads to elevated ROS levels, membrane depolarization, mitochondrial fragmentation, decreased oxidative phosphorylation and ATP production [46,81,82,83].

PTEN is the major negative regulator of the PI3K/Akt signaling pathway [84], and thus, a modulation of its expression or activation has a profound effect on cellular function.

Walker et al., in fact, discovered that inhibition of PTEN promoted neural cell survival, neuroprotection, and neuroregeneration, which in turn promoted myelination of axons through the AKT activation. Actually, PTEN inhibition reduces tissue damage and neuronal cell death and promotes the functional recovery of neurons [85]. After crush injury, deletion of PTEN promotes significant nerve regeneration with enhanced axon regeneration [86]. Therefore, Akt activation may likely play a therapeutic role in neurodegenerative diseases [87,88].

On the other hand, PTEN activation in neurons may have a positive role in neuroprotection [19,89]: its upregulation causes the modulation of the PI3K/Akt signaling pathway, decreasing ROS generation in cells [90]. Deletion of the PTEN gene results in cognitive impairment [91]. Khalil et al. reported that PTEN induced kinase 1 (PINK1) induces mitophagy, promoting neuroprotection in Huntington’s disease [92].

At a systemic level, PI3K induces the activation of NF-κB, and specifically, class I PI3Ks are involved in the transduction pathway of TLRs in immune cells, such as macrophages and dendritic cells. It has been shown that in activated microglia, the Akt activation precedes NF-κB dependent transcription of pro-inflammatory genes [15]. Excessive microglia activation may lead to synaptic loss and neuronal dysfunction.

Activation of different isoforms of the class I PI3Ks plays either positive or negative roles in the production of pro-inflammatory cytokines [93]. Troutman et al. showed that the activation of TLRs can induce the recruitment of class I PI3Ks, which leads to downregulation in NF-κB induced pro-inflammatory cytokines expression in macrophages [93]. On the other hand, Aksoy et al. found that in dendritic cells, loss of functional PI3K decreases TLR4 internalization and relocation to endosomes, leading to a nearby secretion of pro-inflammatory cytokines (IL-6 and IL-12) and a reduction of the production of anti-inflammatory cytokines such as IL-10 and interferon-γ (IFN-γ) [94]. In vitro and in vivo, PI3K switches on the initial signal transduction events downstream of chemoattractant and chemokine G protein coupled receptors (receptors for fMLP, C5a, IL-8, and LTB4), and this leads to the extravasation and migration of innate immune cells, such as neutrophils, monocytes, or eosinophil, during inflammation [95,96,97,98].

Wortmannin and LY294002 are inhibitors of the PI3K/Akt pathway, which prevent ATP from binding to the PI3Ks [99,100]; these inhibitors are cell permeable and low molecular weight compounds, but wortmannin irreversibly inhibits PI3Ks, while LY294002 inhibition is reversible [101]. PI3K inhibitors have been discovered to affect cell growth, proliferation, and survival of cancer cells, as predicted before.

In 2016, Wang and co-workers found that in male C57BL/6 mice ZSTK474, an inhibitor of PI3K had a neuroprotective action because it mitigated cerebral ischemic/reperfusion injury by fostering a beneficial microglial/macrophage phenotype [102].

In addition to synthetic inhibitors, also several natural compounds were involved in PI3K pathway modulation, many of which were flavonoids (see Table 2). The biological actions of flavonoids are due to their antioxidant properties, either through their reducing capacities per se or through their influences on the intracellular redox status [103].

Flavonoids exert their neuroprotective actions by the modulation of intracellular signaling cascades, which control neuronal survival, death, and differentiation, by affecting gene expression or by interactions with mitochondria [104,105].

Many studies have demonstrated that flavonoids inhibit PI3K via direct interactions with its ATP binding site [106,107]. LY294002, one of the most selective PI3K inhibitors, was modelled on the structure of quercetin [108]: they both fit into the ATP binding pocket of the enzyme, but with different orientations [109]. Quercetin inhibits basal Akt phosphorylation at both the regulatory serine 473 and catalytic threonine 308 sites, inactivating it. During neuronal exposure to quercetin, Akt/PKB is inhibited; this leads to extensive caspase-3 activation and subsequent caspase dependent cleavage of Akt/PKB, with subsequent turn off of the major survival signal and the acceleration of apoptotic death [104]. Quercetin, at low concentrations, may activate the MAPK pathway (ERK2, JNK1, and p38); this leads to the expression of survival genes (c-Fos, c-Jun) and defensive genes (phase II detoxifying enzymes; glutathione-S-transferase, quinone reductase), resulting in survival and protective mechanisms, whereas high concentrations stimulate the above depicted pro-apoptotic pathways and ultimate caspase activation [110]. Flavonoids may modulate the PI3K/Akt signaling pathway by the modulation of the expression or the activity of PTEN [111,112].

FLZ, a natural squamosamide derivative from a Chinese herb, has a neuroprotective role because of its capacity to orchestrate PI3K/Akt signal during neuroinflammation. The study of Tai demonstrated that, in LPS induced in vivo and in vitro PD models of inflammation, FLZ was able to inhibit over-activated microglia and protect dopaminergic neurons by the significant inhibition of PI3K/Akt phosphorylation [113].

The cycloartane-type saponin molecule found in roots of *Astragalus* (ASI) is able to inhibit microglia activation, in LPS treated BV2 cells and in an in vivo neuroinflammatory disease model represented by experimental autoimmune encephalomyelitis (EAE) mice. This effect was obtained by inhibition of PI3K and Akt activation, which in turn led to the deactivation of NF-κB, suggesting that ASI modulates PI3K/Akt, acting through glucocorticoid receptors (GRs) as an active ligand [114].

Furthermore, PTEN has been documented to play a critical role in neural functions, whose level has been shown to be reduced in AD brains [115].

PTEN gene expression is modulated by dietary intake of isothiocyanate sulforaphane, such as isothiocyanate derived from broccoli [116]. Dietary exposure to the soy isoflavone such as genistein also induces PTEN expression at physiologically relevant concentrations [117]. Phytoestrogen exposure might result in an increase in PTEN expression and the subsequent decrease in cellular responses including Akt phosphorylation. Fish oil rich in polyunsaturated fatty acids may induce the PTEN expression by activation of the peroxisome proliferator activated receptor (PPAR) [118,119], which also attenuates neuron cellular damage after brain ischemia and plays an important role in the activation of anti-apoptotic signaling [120].

By contrast, a high fat diet attenuates neuroprotection because of decreased activation of Akt signaling [121].

Several lines of evidence have indicated that inhibition of the LPS activated microglial PI3K/Akt pathway leads to a diminished level of proinflammatory factors [15,122]. Studies in LPS stimulated microglial cells demonstrated that curcumin attenuates the expression of TNF-α, IL-6, and IL-1β through the suppression of PI3K/Akt mediated activities [57]. By contrast, Tarassishin and coworkers [70] showed that the PI3K/Akt pathway is also involved in the promotion of the beneficial M2 microglial polarization state. The activation of this signaling cascade suppresses the M1 state and enhances the M2 state after IRF3 stimulation. The authors suggested that inhibition of the pro-inflammatory genes (M1 markers) correlates, at least in part, to the induction of the anti-inflammatory factors (M2 markers) such as IL-1ra and IL-10 and that this process is mediated by the PI3K/Akt pathway. The discrepancies in the effects of the activation of the intracellular pathways in microglial cells may depend on the different input signals [70].

Morin, a flavonoid, exerts anti-inflammatory effects by downregulating MAPK and PI3K/Akt signaling pathways and upregulating the anti-inflammatory PKA/CREB and Nrf2/HO-1 pathways in microglia [75]. Furthermore, TGF-1α, an anti-inflammatory molecule, protects brain by repressing the overactivation of microglial cells via inhibition of PI3K and its downstream signaling molecules [123].

Remarkably, in neuroinflammation, not only glial cells, but also neurons are involved, such as hippocampal ones that can contribute by releasing TNF-α and IL-1β via TLR4 mediated PI3K/Akt/NF-ĸB signaling [124].

Presenilins may play an essential role in signaling pathways that are critical for the pathogenesis of AD by the regulation of the hypoxia inducible factor 1α [125]. Presenilins are responsible for the cleavage of the amyloid precursor protein to form amyloid-β. Phosphorylation of presenilin 1 leads to activation of the PI3K/Akt survival signaling [75].

In Table 2, different compounds able to activate/inhibit PI3K are listed.

## 6. Concluding Remarks

**Figure 2:**
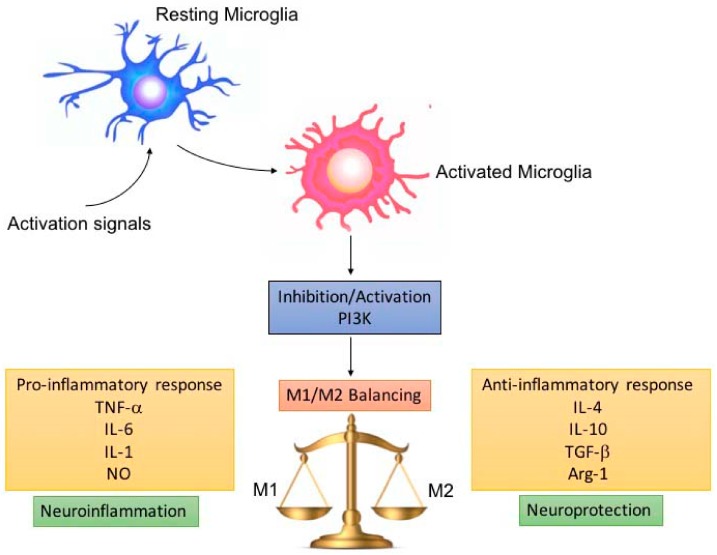
M1/M2 microglia balance depending on PI3K inhibition/activation. Pro-inflammatory microglia (M1) release pro-inflammatory cytokines, which promote neuroinflammation, depending on damage severity and duration. Microglia are able to switch to the anti-inflammatory (M2) phenotype, leading to neuroprotection. The switch and balancing between the M1/M2 phenotype can be regulated by modulating PI3K inhibition/activation.

In this review, the crucial role played by PI3K during neuroinflammatory processes emerged and how its modulation (activation/inhibition) influenced the balance of the microglial pro- and anti-inflammatory responses, which in turn was essential in the neuroprotective outcome after the neuroinflammatory phase (Figure 2).

In fact, this balance is of fundamental importance in the transition from the neuroinflammatory phase, aimed at the elimination of neurotoxic insults, to the reparative and anti-inflammatory events that restore brain homeostasis.

PI3K mediated signaling coordinates a variety of complex events regulating inflammatory responses of activated microglia that, in turn, may influence neuronal survival, so regulation of PI3K enzymes may have implications in neuronal physiology in healthy and disease states. Therapeutic intervention modulating PI3K activation could not only contribute to the prevention of neurodegenerative diseases, but also reduce their progression. Therefore, the exploitation of PI3Ks role in activated microglia responses may also likely represent a further promising area for drug development, both in neuroimmunotherapy and modulation of inflammation induced brain degeneration.

## Figures and Tables

**Table 1:** Tissue distribution of PI3K isoforms.

PI3k	Expression	Ref
**p110 α**	Ubiquitous	[14,23]
**p110 β**	Ubiquitous	[14,23,24]
**p110 δ**	Immune cells, Neurons and Microglia, Spleen, Platelets, Endothelial Cells	[25]
**p85 α**	Ubiquitous	[24]
**p55 α**	Brain, Muscle	[26]
**p50 α**	Liver, Kidney, Brain, T Cells	[25]
**p85 β**	Ubiquitous	[24,26]
**p55 γ**	Brain, Testis, Liver, Muscle, Fat, Spleen	[24]
**p110 γ**	Immune Cells, Heart, Pancreas, Liver, Skeletal Muscle	[25,27]
**p101**	Immune Cells, Mast Cells	[26,28,29]
**p84/p87**	Immune cells, Mast Cell, Heart	[26,28,29]
**pI3k-C2**	Ubiquitous	[30]
**pI3k-C2β**	Ubiquitous	[30]
**pI3k-C2 γ**	Liver, Prostate, Breast, Salivary Gland	[31]
**Vps34**	Ubiquitous	[32]
**Vps15**	Ubiquitous	[26]

**Table 2:** Compounds involved in the activation/inhibition of the PI3K pathway.

Compounds	Nature	Function	Ref.
**Myricetin**	Flavonoid	Inhibition	[106]
**Baicalein**	Flavonoid	Inhibition	[126]
**Apigenin**	Flavonoid	Inhibition	[27]
**Morin**	Flavonoid	Inhibition	[74]
**Quercetin**	Flavonoid	Inhibition	[127]
**Puerarin**	Flavonoid	Activation	[128]
**Hesperidin**	Flavonoid	Activation	[129]
**EGCG**	Polyphenol	Inhibition	[130]
**Curcumin**	Polyphenol	Inhibition	[57,88]
**Wortmannin**	A fungal metabolite	Inhibition	[131]
**Irisin**	Hormone	Activation	[132]
**Insulin**	Hormone	Activation	[133]
**BDNF**	Brain-derived neurotrophic factor	Activation	[131]
**PDGF**	Platelet-derived growth factor	Activation	[43]
**EGF**	Epidermal growth factor	Activation	[43]
**TNFR2**	75-kDa TNF receptor type II	Activation	[134]
**TGF-1α**	Pleiotropic cytokine	Inhibition	[135]
**FAM3A**	Cytokine-like gene family	Activation	[136]
**NSE**	Neuron specific enolase	Activation	[137]
**6-MP**	Thiopurine	Inhibition	[137]
**LY294002**	Inhibitor	Inhibition	[138]
**ZSTK474**	Class I PI3K inhibitor	Inhibition	[132]
